# The Prosensory Function of Sox2 in the Chicken Inner Ear Relies on the Direct Regulation of *Atoh1*

**DOI:** 10.1371/journal.pone.0030871

**Published:** 2012-01-23

**Authors:** Joana Neves, Masanori Uchikawa, Anna Bigas, Fernando Giraldez

**Affiliations:** 1 CEXS, Universitat Pompeu Fabra, Parc de Recerca Biomèdica de Barcelona (PRBB), Barcelona, Spain; 2 Graduate School of Frontier Biosciences, Osaka University, Osaka, Japan; 3 Program in Cancer Research, IMIM-Hospital del Mar, Parc de Recerca Biomèdica de Barcelona (PRBB), Barcelona, Spain; Texas A&M University, United States of America

## Abstract

The proneural gene *Atoh1* is crucial for the development of inner ear hair cells and it requires the function of the transcription factor Sox2 through yet unknown mechanisms. In the present work, we used the chicken embryo and HEK293T cells to explore the regulation of *Atoh1* by Sox2. The results show that hair cells derive from Sox2-positive otic progenitors and that Sox2 directly activates *Atoh1* through a transcriptional activator function that requires the integrity of Sox2 DNA binding domain. *Atoh1* activation depends on Sox transcription factor binding sites (SoxTFBS) present in the *Atoh1* 3′ enhancer where Sox2 directly binds, as shown by site directed mutagenesis and chromatin immunoprecipitation (ChIP). In the inner ear, *Atoh1* enhancer activity is detected in the neurosensory domain and it depends on Sox2. Dominant negative competition (Sox2HMG-Engrailed) and mutation of the SoxTFBS abolish the reporter activity in vivo. Moreover, ChIP assay in isolated otic vesicles shows that Sox2 is bound to the *Atoh1* enhancer in vivo. However, besides activating *Atoh1*, Sox2 also promotes the expression of *Atoh1* negative regulators and the temporal profile of *Atoh1* activation by Sox2 is transient suggesting that Sox2 triggers an incoherent feed-forward loop. These results provide a mechanism for the prosensory function of Sox2 in the inner ear. We suggest that sensory competence is established early in otic development through the activation of *Atoh1* by Sox2, however, hair cell differentiation is prevented until later stages by the parallel activation of negative regulators of *Atoh1* function.

## Introduction

The inner ear provides the brain with accurate information on mechanical perturbations that result in the perception of sound and balance. Mechano-electrical transduction is initiated by the highly specialized hair cells, which transmit electrical signals to the primary afferent neurons that convey this information to the brain. There is good evidence that hair cell fate depends on the function of the proneural factor *Atoh1*, that behaves as a master gene for hair cell differentiation [1], [2], [3]. *Atoh1* is an Helix-Loop-Helix (HLH) transcription factor regulated through a positive autoregulatory loop that maintains its expression in the sensory precursors [4], [5], and through the negative regulation of other HLH proteins that prevent *Atoh1* expression and function [5], [6], [7], [8], [9], [10], [11], [12]. Yet, the molecular mechanisms underlying the onset of *Atoh1* expression remain obscure.

Sox2 is a High Mobility Group (HMG) box domain transcription factor that belongs to the B1 subfamily of Sox proteins [13] and it behaves as a transcriptional activator [14]. Sox2 shows two seemingly contradictory functions in the developing inner ear. On one hand, it is expressed in neurogenic and sensory progenitors [15], [16], [17] and it is necessary for hair cell development [18]. Misexpression of *Sox2* results in an increased number of neurons and ectopic hair cells [19], [20]. On the other hand, Sox2 counteracts Atoh1 function and prevents hair cell formation when over-expressed in sensory precursors [21]. This is reminiscent of the function of SoxB1 genes in the Central Nervous System (CNS), where they promote neural competence but prevent neuronal differentiation [22], [23], [24]. Since neural commitment depends ultimately on the expression of proneural genes, the general question arises as to how Sox2 regulates proneural gene function.

In the present work, we show that Sox2 directly activates *Atoh1* transcription in the early otic vesicle, providing a molecular mechanism for the prosensory function of Sox2 in the inner ear. Besides, we found that Sox2 regulates *Atoh1* through an incoherent logic that promotes the expression of both *Atoh1* and *Atoh1* negative regulators. We suggest that as a result of this dual interaction, otic progenitors are committed to sensory fate early in development, but their differentiation deferred until later stages.

## Methods

### Plasmids and constructs

The NOP2-EGFP contains EGFP under the control of *Sox2* nasal and otic enhancer [25]. Atoh1enh-BG-EGFP and Atoh1enh-BG-ZA (J.Johnson Lab, Dallas, USA) contain the 1,4 kb *Atoh1* enhancer region 5′ to the β-globin basal promoter, the *EGFP* or *lacZ* coding regions, respectively, and SV40 polyadenylation sequences [4]. The Atoh1enhmut-BG-EGFP and Atoh1enhmut-BG-ZA are similar to Atoh1enh-BG-EGFP and Atoh1enh-BG-ZA but each contains three point mutations in the SoxTFBS (see below, site-directed mutagenesis). Either pCMV/SV1-cSox2 or mSox2pCDNA3 (P. Scotting lab, Nottingham, UK) were used for Sox2 misexpression in vivo and in vitro with similar results. The pCMV/SV1-cSox2HMG-VP16/Engrailed has the C-terminal domain of Sox2 coding region (aa 184 till C-terminal) replaced by the VP16 trans-activator domain/Engrailed repressor domain. The pCMV/SV1-cSox2ΔHMG has the HMG domain (aa 3–202) removed. pDsRed (Clontech), pCIG-EGFP (Elisa Marti, Barcelona, Spain) and pCMV-luciferase (R.Perona, Madrid, Spain) were used as controls for electroporation domains and cell transfection levels.

### Site directed mutagenesis

The mutated reporter constructs Atoh1enhmut-BG-EGFP and Atoh1enhmut-BG-ZA were generated using the QuickChange® Site-Directed Mutagenesis Kit (Stratagene). Briefly, mutually complementary primers (Invitrogen, sequence available upon request) aligning with the region of the *Atoh1* enhancer containing the SoxTFBS were designed according to the manufacturer's instructions to create three point mutations. The mutated reporter construct was replicated in a PCR reaction and the parental DNA digested with DpnI. Undigested mutated constructs were amplified in bacterial hosts and sequenced to detect the insertion of the desired mutation before using in subsequent functional assays.

### Chicken (Gallus gallus) embryos and in ovo electroporation

Fertilized hens' eggs (Granja Gibert, Tarragona, Spain) were incubated at 38°C for designated times and embryos were staged according to Hamburger and Hamilton [26]. HH12-14 chicken embryos were electroporated *in ovo* with the desired vector (1 µg/µl, for *Sox2* expression vectors, 1,5 µg/µl for *Atoh1* reporter; 2 µg/µl for *Sox2* reporter) mixed with fast green (0.4 µg/µl) that were injected onto the otic cup by gentle air pressure through a fine micropipette. Square pulses (8 pulses of 10 V, 50 Hz, 250 ms) were generated by an electroporator Square CUY-21 (BEX Co., LTd, Tokiwasaiensu, Japan). Focal electroporation of HH20-21 otic vesicles was performed *in ovo*, using a method modified from Chang et al. [27].

### HEK293T cell transfection

HEK293T cells were cultured in DMEM supplemented with glutamine, antibiotics and 10% fetal bovine serum. Before transfection, cells were cultured in serum and antibiotics-free medium. For transfection, the DNA was mixed with Polyethylenimine 1 mg/ml (PEI, Polysciences Inc, PA, USA) at the ratio of 4 µl of PEI/µg of DNA, incubated twenty minutes at room temperature and finally added to the cell culture. For *Atoh1* enhancer activity assays, 1 µg of *Sox2* expression vector (or Sox2HMG-VP16 or Sox2ΔHMG) was co-transfected with 0,5 µg of Atoh1eh-BG-ZA and 0,2 µg of pCMV-Luciferase for βgal activity assays, or 0,5 µg of Atoh1eh-BG-EGFP and 0,2 µg of pDsRed for direct fluorescence assays. For Western blot and qRT-PCR analysis, 1 µg of *Sox2* expression vector was co-transfected with 0,2 µg of pCIG-EGFP.

### Immunohistochemistry

Embryos were sectioned and processed according to Neves et al. [15]. Primary antibodies were: α-Jag1 rabbit polyclonal (Santa Cruz Biotechnology, Inc, sc- 8303, H-114,1∶50); α-GFP mouse monoclonal (Invitrogen, 1∶400); α-GFP rabbit polyclonal (Clontech, 1∶400); α-Sox2 goat polyclonal (Santa Cruz Biotechnology, Inc., sc-17320, Y-17, 1∶400); α-MyoVIIa mouse monoclonal (DSHB, 138-1, 1∶300); α-Islet1 mouse monoclonal (DSHB, 39.4D5, 1∶400) and α-HCA mouse monoclonal (gift of Guy Richardson, D10, 1∶500). Secondary antibodies were Alexa Fluor-488, -594 and -568 conjugated and HRP-conjugated anti-goat or anti-rabbit (Dako, 1∶500). HRP staining was developed with DAB substrate (Sigma). Sections were counterstained with DAPI (100 ng/ml, Molecular Probes) and mounted in Mowiol media (Calbiochem). Fluorescence was analyzed in whole embryos and in 20 µm cryostat sections by conventional fluorescence microscopy (Leica DMRB Fluorescence Microscope with Leica CCD camera DC300F). Images were processed with Adobe Photoshop.

### Quantitative real time PCR (qRT-PCR)

Eight to twelve otic vesicles were dissected and total RNA isolated using RNeasy Mini kit (Qiagen). For HEK293T cells, total RNA from 6-well plates was isolated with a standard Trizol extraction (Invitrogen). Retrotranscription of 15 ng (chicken samples) or 1 µg (HEK293T samples) of purified mRNA was used to synthesize cDNA with Superscript III DNA polymerase (Invitrogen) and random primers (Invitrogen). Real time PCR was carried out using SybrGreen master mix (Roche), 1 µl of retrotranscribed cDNA and specific primers sets for each gene (Invitrogen, primer sequences are available upon request), in LightCycler480 (Roche). cGAPDH and hPum1 were used as calibrator genes for chicken and HEK293T samples, respectively. Expression levels of each gene were normalized to the calibrator gene and then referred to the levels in control samples, which were arbitrarily set to 1. Transcription levels were further normalized to co-transfected GFP. Quantitative real-time PCR experiments were performed with cDNA from three independent biological replicates.

### βGal and luciferase enzymatic assays

Protein extracts from cells were prepared using Reporter Lysis buffer (Promega) according to the manufacturer's instructions. For βGal activity, triplicates of each protein extract (10 µl) was mixed with 90 µl βGal staining solution (100 mM PBS, 100 mM MgCl_2_, 4 mg/ml ONPG, 4,5 M βmercaptoethanol) in a 96-well ELISA plate and incubated for 2–20 h at 37°C. βGal activity was determined by the absorbance at 420 nm in a microplate reader (VERSAmax, Molecular Devices, Cape Cod). For luciferase activity, 10 µl of each protein extract was mixed with 20 µl of Luciferase Assay Reagent (Promega) and activity was determined with a Luminescence Microplate Reader (Clarity, BioTek). For each well, βGal activity was normalized for the level of transfection using luciferase activity and then the values in transfected samples were referred to the corresponding control, which was arbitrarily set to 1. Enzymatic activity was measured with protein extracts from three independent biological replicates.

### Western Blot

Protein extracts were prepared using a mild protein extraction buffer (PBS-EDTA 1 mM, Na_3_VO_4_ 100 µm, β Glycerolphosphate 20 mM, PMSF 0,2 mM, 0,5% Triton). Proteins were separated in 12%polyacrylamide gels and transferred to a PVDF membrane (Immobilon-P, Millipore). Membrane was blocked with 5% milk in Tris buffered saline with 0,1% Tween (TBST) and incubated overnight at 4°C with primary antibodies diluted in 1% milk in TBST, with gentle shaking. Membranes were washed with TBST, incubated with secondary antibodies, washed first with TBST and then with TBS, and developed with SuperSignal West Pico Chemiluminescent substrate (Pierce). Primary antibodies were α-Sox2 goat polyclonal (Santa Cruz Biotechnology, Inc, sc-17320, Y-17, 1∶500); α-Atoh1 rabbit polyclonal (Abcam, ab13483, 1∶1000); α-GFP rabbit polyclonal (Clontech, 1∶1000) and α-Tubulin mouse monoclonal (Sigma, 1∶2000). Secondary antibodies were HRP-conjugated donkey anti-goat or anti-rabbit (Jackson ImmunoResearch Laboratories, Inc, 1∶5000) and HRP-conjugated rabbit anti-mouse (Dako, 1∶2000).

### Chromatin Immunoprecipitation (ChIP)

HEK293T cells or dissected otic vesicles were processed for ChIP as previously described [28]. Briefly, formaldehyde cross-linked cell or tissue extracts were sonicated in a Bioruptor (Diagenode), and the chromatin fraction incubated overnight with 5 µg of either Goat IgG (Purified Immunoglobulin, Sigma, I9140) or α-Sox2 goat polyclonal antibody (Santa Cruz Biotechnology, Inc., sc-17320, Y-17) in RIPA buffer, and precipitated with protein A/G-Sepharose (Amersham). Cross-linkage of the co-precipitated DNA-protein complexes was reversed, and DNA was analyzed by qRT-PCR as described above. Primers used to detect the different regions of chromatin are available upon request.

### Results analysis and statistics

qRT-PCR analysis, reporter enzymatic activity and in vitro ChIP assays were performed with three independent biological replicates. In vivo ChIP assays were performed with two independent biological replicates. The results are shown as mean±SE for one typical experiment, and statistical significance was assessed using Students' t test applied to the three independent experiments. p<0,001 is labeled with ***, p<0,005 is labeled with ** and p<0,05 is labeled with *. n.s., non significant.

## Results

### Hair cells and neurons derive from Sox2-positive progenitors

**Figure 1:**
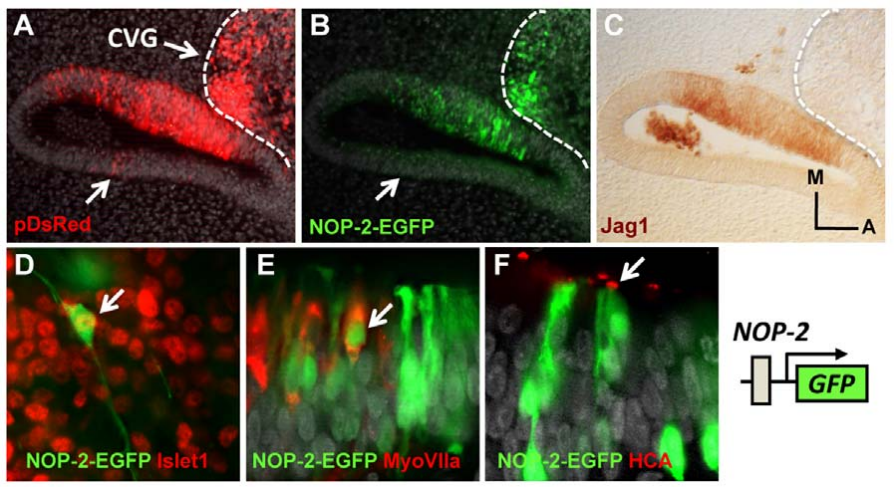
Tracing Sox2-positive progenitors. A–C, Coronal section of an HH22 otic vesicle electroporated with pDsRed (A) and NOP-2–EGFP (B) at HH12 and immunostained for Jag1 (C). The dotted line labels the cochleo-vestibular ganglion (CVG). The arrow indicates an electroporated domain, outside the Jag1-positive region, where the reporter is not active. **D–F**, Detail of the electroporated epithelium showing the co-localization of EGFP driven from the NOP-2 reporter with Islet1 in neurons (D), and with MyoVIIa (E) and HCA (F) in hair cells. Arrows indicate double labeled cells. A, anterior; M, medial.

Previous work suggests that Sox2 promotes the competence to generate neurons and hair cells in the otic vesicle [19], [20]. This predicts that in the embryo, both cell types derive from Sox2-positive progenitors. To analyze this possibility, we electroporated the NOP-2-EGFP in HH12 chicken embryos and followed the fate of the progeny with specific markers. The NOP-2-EGFP construct contains the EGFP reporter gene under the control of a Sox2 enhancer that drives expression specifically in otic and nasal placodes [25]. The stability of EGFP provides a cumulative labeling of cells that expressed Sox2 throughout the experiment and, hence, the lineage of Sox2-expressing progenitors (Fig. 1A–F). In 11 samples, EGFP-positive cells were detected both in the prosensory domain (compare B and C) and in the cochleo-vestibular ganglion (dotted line, B). Neuronal fate of the Sox2 progeny was confirmed by co-labeling with Islet1 antibody (D, n = 4), and that of hair cells by co-labeling with MyoVIIa and Hair Cell Specific (HCA) antibodies (E and F, n = 4). The results indicate that both hair cells and neurons derive from Sox2-positive progenitors.

### Sox2 induces the transcription of Atoh1

**Figure 2:**
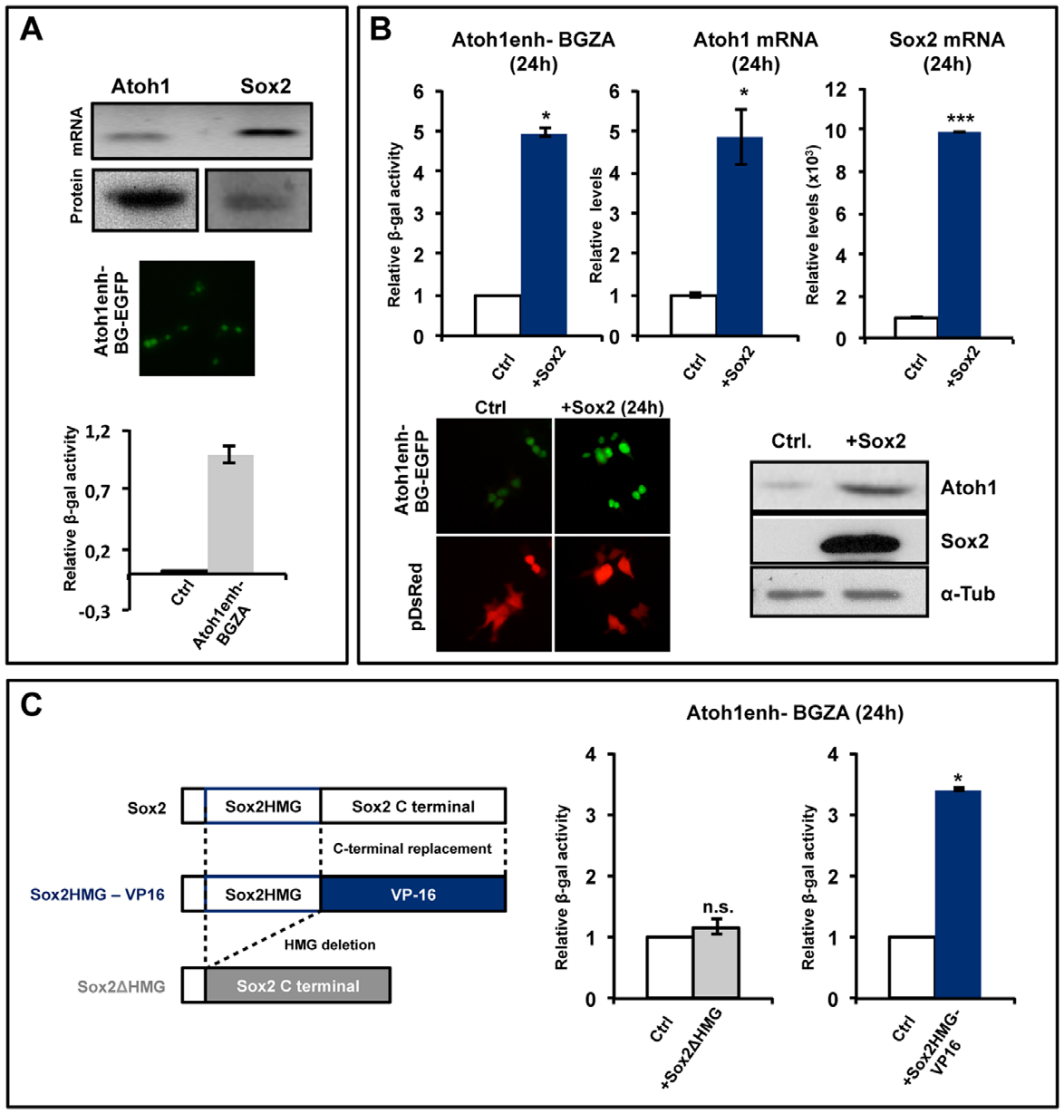
Sox2 induces *Atoh1* expression. A, Endogenous expression of Sox2 and *Atoh1* in HEK293T cells. RT-PCR and Western blot showing the endogenous expression of Sox2 and *Atoh1* mRNA and protein, respectively (top). Direct green fluorescence in HEK293T cells transfected with Atoh1enh-BG-EGFP (middle). βGal activity in protein extracts of HEK293T cells transfected with Atoh1enh-BG-ZA (bottom). **B,** ***Sox2*** **induces** ***Atoh1*** **expression in HEK293T cells.** Relative βGal activity in HEK293T cells co-transfected with *Sox2* and Atoh1enh-BG-ZA one day after transfection (top, left bar diagram). Relative mRNA levels of *Atoh1* and *Sox2* in HEK293T cells transfected with *Sox2* for one day (middle and right bar diagrams). Direct green and red fluorescence in HEK293T cells co-transfected with pDsRed (for transfection level control) and Atoh1en-BG-EGFP (bottom left). Western blot analysis of HEK293T protein extracts one day after *Sox2* transfection showing Atoh1 protein induction (bottom right). Endogenous Sox2 protein levels were too low to be detected in the same blot. All techniques show an induction of *Atoh1* after Sox2 transfection. **C,** ***Atoh1*** **regulation depends on the function of Sox2 as a transcriptional activator.** Structure of the *Sox2* mutant constructs used in the experiment (left, see Methods). Analysis like in Fig. 2B, showing the relative βGal activity in HEK293T cells co-transfected with Atoh1enh-BG-ZA and Sox2ΔHMG (grey) or Sox2HMG-VP16 (blue) (right graph). Deletion of DNA binding domain eliminates the effects on *Atoh1* enhancer activity while Sox2HMG-VP16 reproduces the effects of Sox2.

Hair cell formation depends on the function of the proneural gene *Atoh1* [3], but it is not known which factors regulate the onset of *Atoh1* expression in the ear. Since Sox2 function is required for *Atoh1* expression and hair cell formation, we asked whether Sox2 was able to induce *Atoh1* expression. HEK293T cells were used as a convenient model system for analysis of molecular interactions before testing their biological significance in vivo. HEK293T cells endogenously expressed *Atoh1* and *Sox2* mRNAs and proteins (Fig. 2A upper). Accordingly, *Atoh1* transcriptional activity was detected after transfection with either EGFP or LacZ *Atoh1* reporter constructs (Fig. 2A, middle photograph and bar diagram, respectively). They contain the reporter genes under the control of *Atoh1* enhancer elements that reside 3′ of the *Atoh1* coding sequence and are sufficient to recapitulate the endogenous *Atoh1* expression in several species, including the chicken [4], [29], [30]. Overexpression of *Sox2* increased *Atoh1* enhancer reporter activity as measured either by βGal activity on cell extracts (Fig. 2B, left bar diagram) or by EGFP fluorescence (Fig. 2B, photographs on the bottom left), confirming previous observations by Neves et al. [20]. Similarly, *Sox2* transfection resulted in an increase in endogenous *Atoh1* mRNA levels (Fig. 2B, middle bar diagram) and in Atoh1 protein (Fig. 2B, low-right).

Since Sox2 is an activator transcription factor [14], the effects of Sox2 on *Atoh1* transcription should be dependent on both DNA-binding and transcriptional activator function. HEK293T cells were co-transfected with the *Atoh1* reporter and with either Sox2HMG-VP16 or Sox2ΔHMG (Fig. 2C, left diagram). The Sox2ΔHMG lacks the DNA binding domain and its co-transfection had no effect on *Atoh1* reporter activity (Fig. 2C, grey bar). This shows that the regulation of *Atoh1* requires the binding of Sox2 to DNA. The Sox2HMG-VP16 construct contains the Sox2 DNA binding domain fused to a potent trans-activator domain. The co-transfection with Sox2HMG-VP16 reproduced the effects of *Sox2* on *Atoh1* (Fig. 2C, blue bar).

These experiments show that Sox2 is able to induce *Atoh1*, that this depends on the function of Sox2 as an activator transcription factor, and that it requires Sox2 binding to DNA.

### Sox2 directly binds to the Atoh1 enhancer

**Figure 3:**
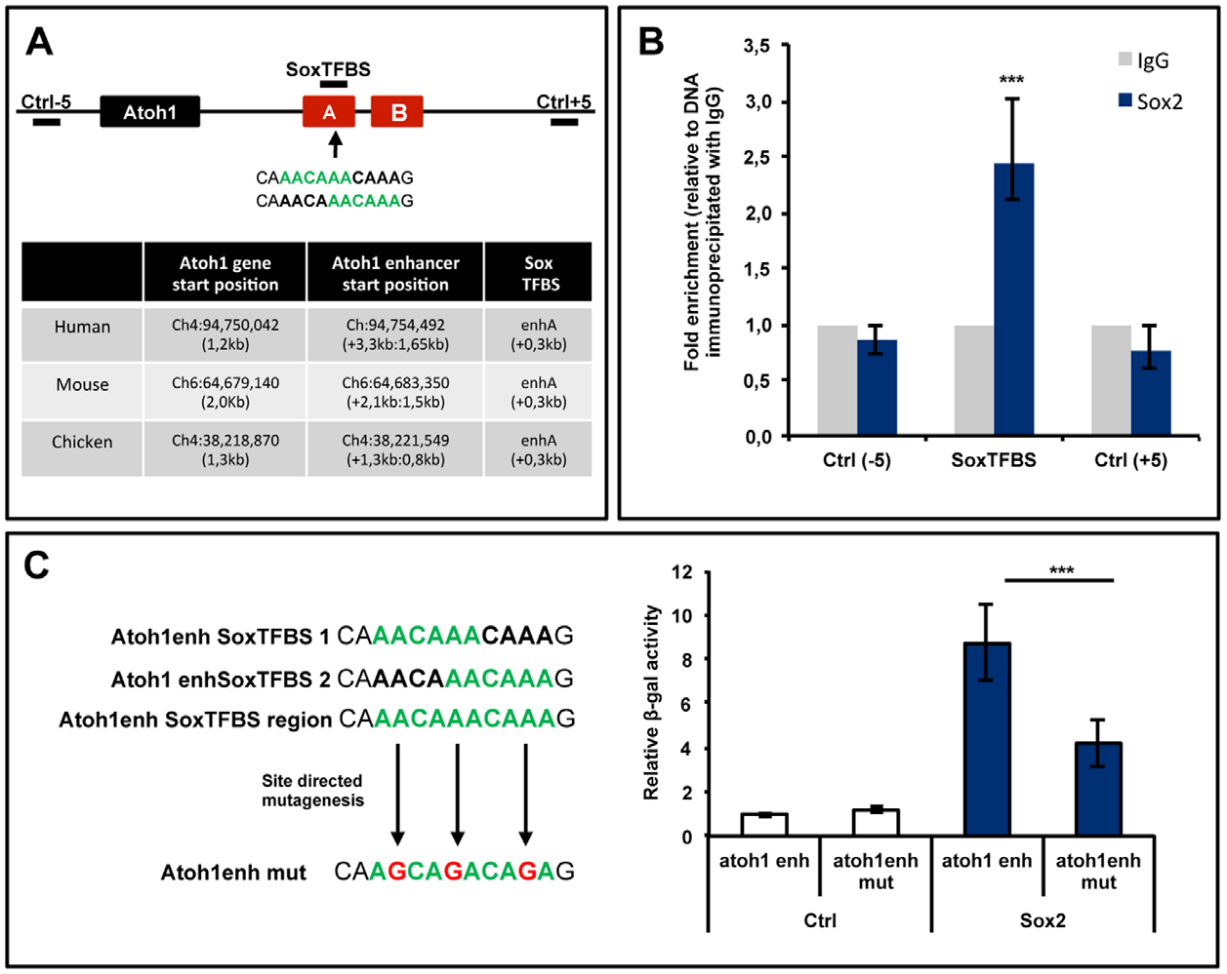
A, *In silico* analysis of the 3′ *Atoh1* enhancer. *Atoh1* locus and regulatory sequences as described in Helms et al. (2000). Arrow indicates the location of the consensus SoxTFBS in the 3′ end of the enhancer sequence A. Green represents the consensus TFBSs and black the immediate flanking sequence. Two lines are used to represent the same sequence in order to undisclosed the two overlapping sites. +5 and −5 label the regions used as controls in the ChIP experiment. The table summarizes the location of *Atoh1* gene and the 3′ enhancer in three different species and the conserved location of the SoxTFBS. **B, ChIP assay in HEK293T cells.** The bar diagram shows the relative amount of chromatin precipitated with Sox2 (blue) with respect to IgG (grey) containing the three different regions of the *Atoh1* locus indicated in the cartoon. Chromatin precipitated with Sox2 was significantly enriched in the SoxTFBS of the *Atoh1* enhancer. **C, Site directed mutagenesis of the SoxTFBS.** Three point mutations were introduced in the *Atoh1* enhancer reporter construct and are indicated in red (left diagram). Bar diagram to the right shows the relative βGal activity in HEK293T cells co-transfected with *Sox2* and Atoh1enhmut-BGZA compared to the native *Atoh1* reporter. The mutated reporter activity was reduced to half.

In order to test the possible binding of Sox2 to the *Atoh1* regulatory regions, the enhancer sequence of *Atoh1* was screened using Transfac database in rVista software and two overlapping Sox Transcription Factor Binding Sites (SoxTFBS) were found. They were conserved among human, mouse and chicken, mapping to the 3′ end of the *Atoh1* enhancer A (Fig. 3A). In order to test the interaction between Sox2 and these binding sites, we performed a ChIP assay. Chromatin from HEK293T cells was immunoprecipitated with a Sox2 antibody and analyzed for the presence of the SoxTFBS with specific primers for the corresponding region of the *Atoh1* enhancer. As controls, we used two regions located 5 kb upstream and downstream of the binding sites. Chromatin precipitated with Sox2 antibody was enriched in the SoxTFBS region of *Atoh1* enhancer when compared to the chromatin precipitated with a goat IgG antibody (Fig.3B). Furthermore this enrichment was specific for this region of the chromatin and not detected in the control sites (n = 3).

Site-directed mutagenesis was used to evaluate whether the induction of *Atoh1* by Sox2 was dependent on binding to these SoxTFBS. Briefly, we introduced three point mutations in the *Atoh1* enhancer reporter construct, which destroys the ability of Sox2 to bind to the conserved SoxTFBS (Fig. 3C, left diagram). Co-transfection of Sox2 with the mutated *Atoh1* enhancer reporter reduced βGal activity to half of the value obtained after co-transfection with the native *Atoh1* enhancer reporter (Fig. 3C, right bar diagram, n = 3, native and mutated reporter activities compared in the same experiment). Interestingly, the mutation of SoxTFBS did not result in the complete reduction *Atoh1* reporter activity to control values. This is likely due to the induction of endogenous *Atoh1* protein after Sox2 transfection (See Fig.2B). *Atoh1* is able to regulate its own expression through the ‘E-box’ in the *Atoh1* enhancer [4], and this site was intact in the construct.

In summary, these experiments show that Sox2 directly binds to the *Atoh1* enhancer and that the regulation of *Atoh1* by Sox2 is, at least in part, mediated by the SoxTFBS present in the *Atoh1* regulatory regions.

### Atoh1 transcriptional activity in the otic vesicle depends on Sox2

**Figure 4:**
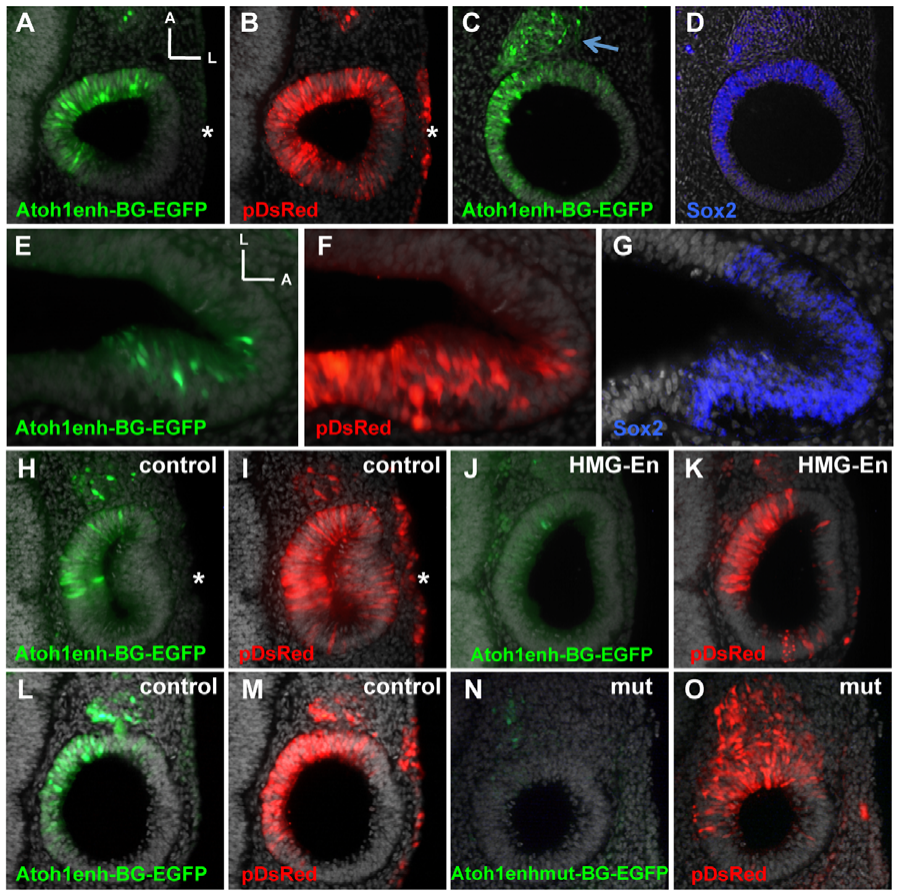
A–D, *Atoh1* reporter activity in the early otic vesicle. Direct green and red fluorescence in coronal sections of a HH17 otic vesicle co-electroporated at HH12 with pDsRed (B) and Atoh1enh-BG-EGFP (A). Coronal section of an HH17 otic vesicle electroporated with Atoh1enh-BG-EGFP (C) at HH12 and immunostained for Sox2 (D). Reporter activity specifically restricted to the anterior-medial aspect of the otic vesicle (compare A and B) and it overlapped with Sox2 expression (compare C and D). Arrow in C indicates reporter activity in the cochleo-vestibular ganglion (CVG), and asterisks indicate the lack of reporter activity in the ectoderm. **E–G,** ***Atoh1*** **reporter activity in early sensory organs.** Coronal section of a crista from an HH24 embryo co-electroporated with *Atoh1* reporter (E) and pDsRed (F) in HH20. The reporter activity restricted to the prosensory domain as shown by Sox2 immunochemistry (G). Figs. 4D and G are HRP staining pseudocolored in blue. **H–O, The** ***Atoh1*** **reporter activity in the otic vesicle is Sox2 dependent.** H–K, Direct red and green fluorescence in coronal sections of a HH17 otic vesicle electroporated at HH12 with the Sox2HMG-Engrailed (J, K, HMG-En) or without (H, I, control). Embryos were co-electroporated with pDsRed and Atoh1enh-BG-EGFP. Green fluorescence derived from the reporter is lost in the presence of Sox2HMG-Engrailed. As above, asterisks indicate that the enhancer was silent in the ectoderm. L–O, Direct red and green fluorescence in coronal sections of a HH17 otic vesicle electroporated at HH12 with pDsRed and Atoh1enhmut-BG-EGFP (N–O). An equivalent electroporation from the same experiment of the native reporter is shown for comparison (L–M). The mutation of the SoxTFBS resulted in the complete loss of reporter activity. A, anterior; L, lateral.

Sox2 is expressed throughout the neurosensory domain of the otic vesicle [15] and we sought to analyze whether this is able to activate *Atoh1* transcription, by using the Atoh1enh-BG-EGFP reporter in vivo. Otic vesicles were electroporated with this reporter construct together with the tracer pDsRed (Fig. 4A–D). *Atoh1* reporter was active in the otic vesicle but spatially restricted to the anterior-medial domain (Fig. 4A and C, n = 19 otic vesicles), corresponding to the Sox2-positive expression domain (Fig. 4D, n = 10 otic vesicles). Note that electroporated cells in the surface ectoderm and lateral aspect of the otic vesicle remained GFP-negative (asterisk in Figs. 4A–B and H–I). Reporter activity was also detected in the neuroblasts of the cochleo-vestibular ganglion, which is consistent with the previous observation these neurons derive from Sox2-positive progenitors (see Fig.1) and suggests that *Atoh1* transcription is also activated by Sox2 in this type of progenitors (arrows in Fig. 4C). Later in development, the activity of the reporter was restricted to the nascent hair cells within the sensory patches (HH24, Fig. 4E–G, n = 11 otic vesicles).

We next tested whether the observed *Atoh1* reporter activity in the otic vesicle indeed depended on Sox2 (Fig. 4H–O). For this purpose we co-electroporated Atoh1enh-BG-EGFP with Sox2HMG-Engrailed, which suppresses Sox2 function as a dominant negative [23]. This resulted in the suppression of the *Atoh1* reporter activity (Fig. 4H–K) suggesting that the early activation of *Atoh1* transcription is dependent on Sox2. Furthermore, the electroporation of the Atoh1enh-BG-EGFP reporter construct carrying the mutation in the SoxTFBS (Atoh1enhmut-BG-EGFP, see Fig. 3C for mutation) resulted in none (Fig. 4N, n = 17/23 otic vesicles) or very low (n = 6/23 otic vesicles) reporter activity in the otic vesicle. This was evaluated by comparing EGFP expression after electroporation of the native *Atoh1* reporter (Fig. 4L, n = 9 otic vesicles) and the mutated *Atoh1* reporter (Fig.4N, n = 23 otic vesicles) for otherwise equivalent electroporations (pDsRed in Fig. 4M and O). Together, these experiments suggest that Sox2 switches on *Atoh1* transcriptional activity in the early otic vesicle.

**Figure 5:**
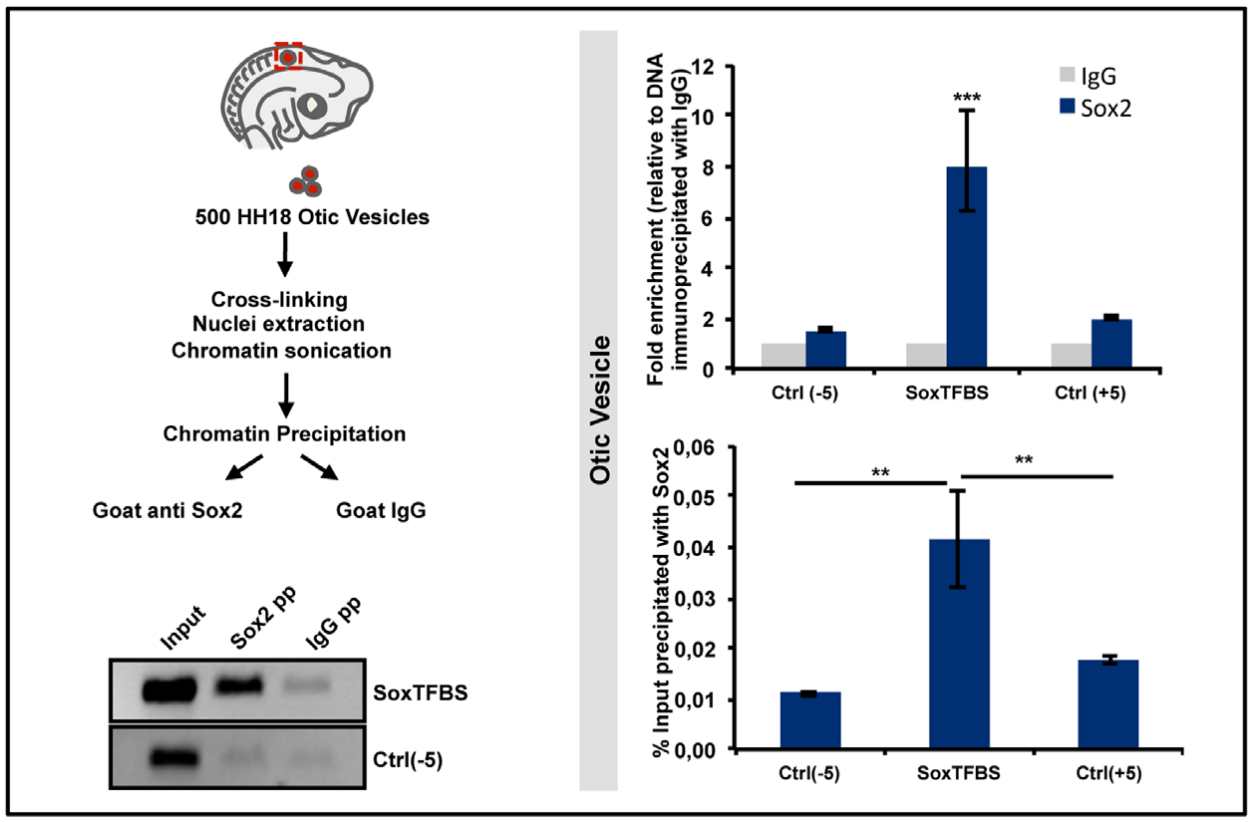
ChIP assay in vivo. Diagram of the experimental design for the ChIP assay in vivo. Otic vesicles (500/experiment) were dissected from HH18 chicken embryos and processed for ChIP as indicated (left). Semi-quantitative RT-PCR of the ChIP assay in vivo (bottom left). Bands represent the fragments amplified with primers for SoxTFBS and for a control region using the input chromatins or the fractions precipitated with Sox2 and IgG as templates. qRT-PCRs of the ChIP assay performed on otic vesicles (right bar diagrams). The bar diagram on the top shows that the chromatin precipitated with Sox2 was significantly enriched in the SoxTFBS of the *Atoh1* enhancer. The bar diagram on the bottom shows the percentage of input chromatin precipitated with Sox2 that contained the three regions analyzed. The fraction of input containing the SoxTFBS of the *Atoh1* enhancer was significantly higher than the ones containing the control regions.

Since *Atoh1* transcription is active in the neurosensory domain of the otic vesicle, one critical question is whether Sox2 binds to the endogenous *Atoh1* enhancer during normal development. In order to test this possibility, we performed ChIP assay in vivo on dissected otic vesicles, as illustrated in Fig. 5 (left). Indeed, there was a significant enrichment in the SoxTFBS region of the *Atoh1* enhancer in the chromatin fraction immunoprecipitated with Sox2 when compared to precipitation with IgG (Fig. 5, upper bar diagram). Furthermore, this enrichment was specific to this region of the genome as the fraction of SoxTFBS precipitated with Sox2 antibody was significantly higher than the fraction of control region precipitated under the same conditions (Fig. 5, lower bar diagram). This demonstrates that in the early otic vesicle, Sox2 is bound to the *Atoh1* enhancer.

In summary, the regulation of *Atoh1* by Sox2 in the otic vesicle relies on the direct binding of Sox2 to the SoxTFBS in the 3′ regulatory region of *Atoh1* enhancer.

### The transient activation of Atoh1 and the induction of inhibitors: an incoherent logic?

**Figure 6:**
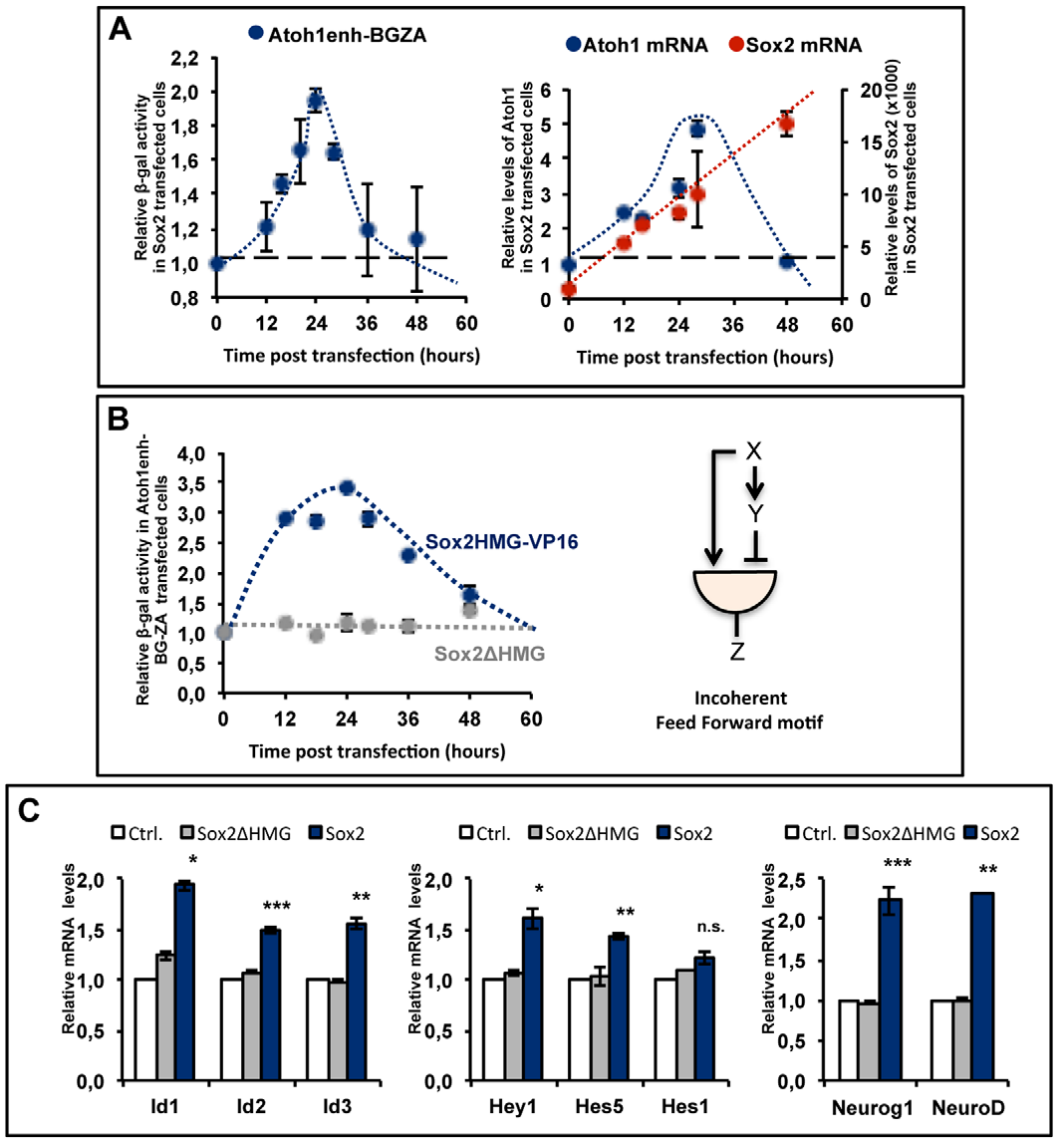
The transient activation of *Atoh1* and the induction of *Atoh1* inhibitors. A, The time course of *Atoh1* activation. Relative βGal activity at different time points in HEK293T cells co-transfected with *Sox2* and Atoh1enh-BG-ZA. For each time point βGal activity is referred as the fold increase with respect to reporter alone, which was arbitrarily set to one (dashed line, left graph). Relative mRNA levels of *Sox2* (red) and *Atoh1* (blue) at different time points, after *Sox2* transfection (right graph). **B, The time course of the HMG-VP16 activation.** Structure of the *Sox2* mutant constructs used in the experiment (left, see Methods). Time course like in Fig. 6A showing the relative βGal activity in HEK293T cells co-transfected with Atoh1enh-BG-ZA and Sox2HMG-VP16 (blue, left graph) or Sox2ΔHMG (grey, left graph). Deletion of DNA binding domain eliminates the effects on *Atoh1* enhancer activity while Sox2HMG-VP16 reproduces the effects of Sox2. Right diagram: Type1 Incoherent Feed Forward loop (I1-FFL, Alon, 2007). The regulator X regulates Y and Z, which is both regulated by X and Y. However, the two arms of the FFL act in opposition and the effect is a transient activation of the target Z. **C,** ***Sox2*** **induces the expression** ***Atoh1*** **negative regulators in the otic vesicle.** **A.** Bar diagram showing the relative mRNA levels of *Id1-3 (left), Hes-Hey (middle)* and *Neurog1 and NeuroD (right)* in otic vesicles transfected with control plasmids (grey bars) or with *Sox2* (blue bars) for one day (*Id-3* and *Hes-Hey*) or two days (Neurog1 and NeuroD). Untransfected otic vesicles (white bar).

The above results suggest that *Atoh1* is directly activated by Sox2 at early developmental stages. However, *Atoh1* expression during pre-differentiation stages is very low or negligible [3], [31]. Several HLH factors like Hes/Hey, Ids, Neurog1 and NeuroD have been involved in the inhibition of Atoh1 expression during otic development [5], [6], [7], [8], [9], [10], [11], [12], [32], and sequence analysis reveals the presence of bHLH binding sites in the *Atoh1* 3′ regulatory regions [4]. Therefore, these factors are potential candidates to counteract the induction of *Atoh1* by Sox2. Besides, Sox2 has been also associated with the negative regulation of *Atoh1* and hair cell formation during ear development [21], a function that is reminiscent of that of SoxB1 genes in CNS development [23], [24]. However, the mechanism behind this seemingly paradoxical situation in which Sox2 is able to both induce and counteract *Atoh1* is unknown. In order to gain insight into this problem we explored further the regulation of *Atoh1* by *Sox2*. A time course analysis of *Atoh1* expression following *Sox2* transfection in HEK293 cells revealed that *Sox2* counteracts its own activator effect on *Atoh1*. *Sox2* transfection induced only a transient activation of *Atoh1* as measured either by *Atoh1* βGal reporter activity or by qRT-PCR analysis of *Atoh1* mRNA (Fig. 6A). The loss of *Atoh1* transcription occurred even though *Sox2* levels increased monotonically throughout the time window of the experiment (Fig. 6A red line in the right graph). Several mechanisms may account for this behavior, but the following data suggest that both activation and inhibition require DNA binding and the transcriptional activator function of Sox2. The co-transfection of Sox2ΔHMG (Fig. 6B, graph, grey) had no effect on *Atoh1* reporter activity, while the co-transfection of Sox2HMG-VP16 (Fig. 6B, graph, blue) reproduced the effects of *Sox2*, both the early up-regulation of *Atoh1* and the delayed return to baseline. This suggests that the inhibition of *Atoh1* by *Sox2* is indirect and requires intermediate factors that change the sign of the activator function of Sox2. Hence, the concurrent activation of inhibitor factors is a plausible explanation. The transient behavior of the *Atoh1* response to *Sox2* is well described by a genetic network where a gene triggers parallel opposing effects on its target (Fig. 6B, right diagram), the Incoherent Feed Forward Loop (I-FFL) as modeled by Allon [33].

The above observations lead us to think that since *Atoh1* expression and hair cell formation in vivo correlate with *Sox2* down-regulation [15], it is possible that *Sox2* cooperates with other signaling pathways that maintain *Atoh1* expression tuned down during pre-differentiation stages. If such a mechanism operates in vivo, one would expect the activation of *Atoh1* inhibitory factors after the overexpression of *Sox2* in the otic vesicle. Therefore, we explored the ability of *Sox2* to induce these factors in the otic vesicle. Indeed, *Sox2* induced the expression of *Id1-3* (Fig. 6C, left bar diagram), *Hes5* and *Hey1* (middle bar diagram) and *Neurog1* and *NeuroD* (right bar diagram) in the otic placode. This indicates that in parallel to *Atoh1* induction, *Sox2* activates and/or modulates the expression of other genes that counteract *Atoh1*. *Neurogenin1* is a direct target of Sox2 in other model systems [34], [35], but it remains to be explored whether this also the case in the otic placode. *Ids* are regulated by BMP signaling [9], and *Hes5* and *Hey1* are down-stream targets of Notch [11], but it is unknown whether Sox2 directly regulates these genes, or if it rather cooperates at other steps in the signaling cascades (see Discussion). In summary, these data suggests that in parallel to the activation of *Atoh1*, *Sox2* induces an incoherent response by promoting the expression of *Atoh1* negative regulators.

## Discussion

### The prosensory function of Sox2: sensory commitment and deferred hair cell differentiation

Throughout evolution, the expression and function of the *Sox2* correlates with the commitment to neural fate [36]. However, *Sox2* prevents proneural gene function and neuronal differentiation [23], [24]. This is also the case during ear development: *Sox2* is necessary for sensory fate specification [18], and the misexpression of *Sox2* results in increased number of neurons and ectopic hair cells [19], [20]. However, *Sox2* shows also an antagonistic function with *Atoh1* that results in the prevention of hair cell differentiation [21]. The aim of this work was to shed light on the mechanism behind this dual function.

The results show that, both in vitro and in vivo, Sox2 is able to directly activate *Atoh1* transcription by binding to the SoxTFBS in the 3′ *Atoh1* enhancer region, as shown by the functional experiments with the mutated reporter and by ChIP analysis. In the early otic vesicle, ChIP assay reveals that Sox2 is bound to the 3′ *Atoh1* enhancer and, moreover, the mutation of the SoxTFBS in the 3′ regulatory region of *Atoh1* suppresses the activity of the enhancer in the otic vesicle. This suggests that *Atoh1* transcription is switched on early in otic development, well before hair cell differentiation, and that Sox2 may be one of the factors involved in the initiation of *Atoh1* expression. Interestingly, this inductive function seems not to be conserved in non amniotes where Sox2 has been reported to have a rather permissive role in respect to Atoh1 [37].

However, during ear development, *Atoh1* is not upregulated until differentiation stages [3], [31], suggesting that the initial induction of *Atoh1* transcription is prevented, in parallel, by specific mechanisms that result in the appropriate timing of hair cell differentiation. Therefore, *Atoh1* expression in neurosensory progenitors would be under the regulation of both activator factors (Sox2, present work) and repressor factors (see below). Bivalent states are characterized by the simultaneous presence of active and repressed chromatin markers in the gene regulatory regions. This is characteristic of many genes involved in cell commitment and pluripotency and it has been reported for the *Atoh1* gene in neural cell lines [38], and in mammalian otic sensory progenitors (Z. Stojanova, T. Kwan and N. Segil, personal communication).

Several factors may account for the prevention of *Atoh1* up-regulation before the stages of hair cell differentiation. Like *Atoh1*, *Neurog1* and *NeuroD* are also bHLH proneural genes of the *Atonal* family, but they drive neurogenesis in the otic vesicle [19]. *Neurog1* inhibits *Atoh1* expression in the inner ear [5] and recent data show that the knock-out of *NeuroD* (a down-stream target of *Neurog1*) generates heterotopic and precocious activation of *Atoh1* and hair cell fates [8]. It is particularly striking that in the cochleo-vestibular ganglion of *NeuroD* null mice, there is a significant number of ectopic hair cell-like cells expressing high *Atoh1* [8]. This is consistent with our observation that *Atoh1* transcriptional activity is found in normal neuroblasts and suggests that unless selectively suppressed, the initial state of neurosensory progenitors is indeed multipotent for both neuronal and hair cell phenotypes. It is worth noting that *Neurog1* and *NeuroD* are transcriptional activators, so the mechanism by which they are able to inhibit *Atoh1* is unclear. In contrast, other HLH genes, like *Hes/Hey* and *Id* family members, are known transcriptional repressors [39], [40], [41], [42]. They are expressed in otic progenitors and their function is associated with the prevention of *Atoh1* function and premature differentiation [7], [9], [10], [11], [12], [43]. In fact, the *Atoh1* enhancer contains a series of bHLH binding sites, which may account for the negative regulation exerted by these genes [4]. Taken together, these factors exert multiple and diverse functions in neural development, but they share a common inhibitory action on *Atoh1* that results in the maintenance of the undifferentiated state of neurosensory progenitors.

### Sox2 activation of Atoh1 inhibitors: an incoherent loop?

Our results show that Sox2 induces the expression of several of the above mentioned inhibitory factors. Although most of them are under the control of specific signaling pathways, Sox2 is nevertheless able to promote their expression. This indicates that Sox2 operates with an incoherent logic with respect to *Atoh1*: it both activates *Atoh1* and promotes its inhibition. Several network motifs have been studied by Alon [33] as a set of recurrent gene regulation patterns that result in predictable functional behaviors. The activation of *Atoh1* by Sox2 fits well with the so-called Type1 Incoherent Feed Forward Loop (I1-FFL) in which the two arms of the FFL act in opposition. The result is a transient target gene activation, with amplitude and timing dependent on the thresholds and time constants of the individual interactions, while the final steady-state level depends on the strength of the inhibition [33]. This type of model predicts well the transient nature of the response of *Atoh1* in the presence of continuously increasing concentrations of *Sox2* mRNA in vitro. Indeed, the fact that the same behavior is induced by the Sox2-HMG VP16 construct indicates that the decay must be induced by intermediate factors that change the sign of the original signal. In our case, Sox2 directly activates *Atoh1* transcription but, on the other hand, Sox2 also up-regulates several inhibitors of *Atoh1* that include *Neurog1*, *NeuroD*, *Hes/Hey* and *Id* genes. This probably causes a balance between activation and inhibition that results in the observed profile of transient activation and steady-state down-regulation of *Atoh1*. This molecular interaction offers a simple explanation for the intriguing dual effects of Sox2: the induction of neural competence and prevention of differentiation. Further experiments will be required to demonstrate the detailed kinetics and the modulation of this genetic network and to describe the detailed mechanisms by which Sox2 modulates the expression of *Atoh1* inhibitors.

*Neurog1* has been previously described as a direct target of Sox2 in neural crest cells [35] and recent work suggests that this may the case also in the inner ear [34]. This provides support to the operation of a 1I-FFL in which Sox2 directly regulates *Atoh1* and also its negative regulator *Neurog1*. However, it remains to be proven that this direct interaction operates in vivo in the otic vesicle. But nevertheless, the suggestion that the direct regulation of *Atoh1* may extend to *Neurog1*, provides an interesting model for the function of Sox2 in the specification of the neurosensory competence of the otic placode, and the sequential generation of neurons and hair cells (see below).

The other *Atoh1* inhibitors regulated by *Sox2* are *Hes5, Hey1*, and *Id1-3*. They have never been described as primary *Sox2* targets, and their regulation during inner ear development is mainly dependent on non-autonomous signaling. Although we cannot exclude the possibility that Sox2 regulates them directly, it is likely that Sox2 cooperates with the signaling pathways that regulate their expression. The regulation of *Hes5* and *Hey1* in the ear is mostly Notch-dependent [11]. *Sox2* misexpression does not affect the expression of Notch ligands in the ear [21]. But in the otic vesicle Sox2 does result in the induction of *Notch1* (Neves et al., unpublished data), and *Notch1* has been identified as a direct target of Sox2 in the retina [44]. On the other hand, *Id* genes are regulated by BMP signaling in the inner ear [9]. Apart from *Id*s, *Sox2* electroporation up-regulates several elements of the BMP pathway that are upstream *Id* transcription. This includes the Smad Interacting Protein 1 (SIP1, Neves et al., unpublished data), which has been identified as a potential Sox2 target by *in silico* analysis [45]. Taken together, the data suggest that unlike *Atoh1* or *Neurog1*, Sox2 may regulate these other inhibitors by interacting with the signaling pathways that regulate their expression.

### Neurosensory competence and the sequential generation of neurons and hair cells in the inner ear

The problem of cell fate specification is central to neural development. How do different cell types with defined phenotypic characteristics originate from multipotent progenitors? The functional unit of the ear consists of three elements of neural origin: the mechano-transducing hair cells, the supporting cells, and the primary afferent neurons. All three elements derive from the neurosensory competent domain of the otic vesicle and their development follows a stereotyped spatial and temporal pattern, with neurons being specified prior to hair cells [46], [47], [48]. Neuronal fate is specified by the expression of the proneural genes *Neurog1* and *NeuroD* [6], [49], [50]. Sensory fate specification occurs after neurogenesis, and commitment to the sensory fate is associated with the expression of *Atoh1* [1], [3].

The observation that both neurons and hair cells derive from Sox2-positive progenitors fits well with the idea of the common origin of both cell types, as suggested by viral and genetic tracing [5], [51]. How does Sox2 specify this dual competence in the otic progenitors? Sox2 is able to induce the expression of proneural genes *Neurog1, NeuroD* and *Atoh1* [34 and present work], which would be sufficient, in principle, to specify neuronal and hair cell fates. But the question then is how these fates are sorted out, and why hair cell fate is delayed with respect to neuronal fate. One possibility is that Sox2 establishes neurosensory competence early in development, by the activation of the major proneural genes *Neurog1* and *Atoh1*. However, the down-regulation of *Atoh1* by *Neurog1* and *NeuroD* would allow neurogenesis but not hair cell differentiation. Cell fate decisions would thus depend on selective repression of the initial neurosensory potential, rather that the temporal acquisition of new properties. It is not until Sox2 is counteracted that *Atoh1* expression would be permitted in hair cells, but not in supporting cells. Daudet and co-workers have recently shown that *Sox21* is expressed during hair cell differentiation and that it is able to inhibit Sox2 expression (N. Daudet, personal communication). A similar interaction between Sox2 and Sox21 was described in the neural tube [52].

In summary, Sox2 promotes sensory fate in the otic vesicle by direct binding to *Atoh1* regulatory sequences. However, *Atoh1* activation is deferred and *Atoh1* up-regulation and hair cell differentiation do not occur until later developmental stages. One possible explanation for this dual effect is that Sox2 triggers an incoherent response that results in a steady-state inhibition of *Atoh1*. This would provide a simple explanation for the dual function of Sox2 in neural development, i.e.: promotion of neural competence and suppression of differentiation.
